# Rapid genomic changes in allopolyploids of *Carassius auratus red var.* (♀) × *Megalobrama amblycephala* (♂)

**DOI:** 10.1038/srep34417

**Published:** 2016-10-05

**Authors:** Qinbo Qin, Zhengfa Lai, Liu Cao, Qiong Xiao, YuDe Wang, Shaojun Liu

**Affiliations:** 1 Key Laboratory of Protein Chemistry and Fish Developmental Biology of Education Ministry of China, College of Life Sciences, Hunan Normal University, Changsha 410081, China

## Abstract

To better understand genomic changes in the early generations after polyploidisation, we examined the chromosomal consequences of genomic merger in allotetraploid hybrids (4 nF_1_) (AABB, 4n = 148) of *Carassius auratus red var.* (RCC) (AA, 2n = 100) (♀) × *Megalobrama amblycephala* (BSB) (BB, 2n = 48) (♂). Complete loss of the paternal 5S rDNA sequence and the expected number of maternal chromosomal loci were found in 4 nF_1_, suggesting directional genomic changes occurred in the first generations after polyploidisation. Recent studies have reported instability of newly established allotetraploid genomes. To assess this in the newly formed 4 nF_1_ genome, we performed fluorescence *in situ* hybridisation on an allotetraploid gynogenetic hybrid (4 nG) (AABB, 4n = 148) and an allopentaploid hybrid (5 nH) (AABBB, 5n = 172) from 4 nF_1_ (♀) × BSB (♂) with 5S rDNA gene and centromere probes from RCC, the original diploid parent. The expected numbers of maternal chromosomal loci were found in 4 nG, while chromosomal locus deletions and chromosome recombinations were detected in 5 nH. These observations suggest that abnormal meiosis did not lead to obvious genomic changes in the newly established allotetraploid genomes, but hybridisation with the original diploid parent resulted in obvious genomic changes in the newly established allotetraploid genomes, as was found for the maternal genome.

Polyploidisation is a significant evolutionary process that results in rapid speciation^1^,^2^,^3^,^4^,^5^,^6^. Many diploids species are actually ancient polyploids that have undergone diploidisation. Recent studies, mostly in plants, suggested that allopolyploid formation could induce various types of genomic changes, including directional sequence elimination, random structural changes, and chromosome structure^7^,^8^,^9^,^10^,^11^,^12^,^13^. Importantly, a variety of genomic changes has been shown to result in diploid-like chromosome pairing^14^,^15^,^16^,^17^. Although the potential contribution of genomic changes to the evolutionary success of polyploidy has been widely recognised, virtually no information is available on how newly established genomes have evolved after polyploidisation.

Because sex determination and development are disrupted by polyploidisation, polyploid species are common in plants, but rarely successful in animals^18^,^19^,^20^. In our previous study, fertile allotetraploid hybrids (4 nF_1_) (AABB, 4n = 148) were successfully obtained in the first generation of *Carassius auratus red var.* (RCC) (AA, 2n = 100, ♀) × *Megalobrama amblycephala* (BSB) (BB, 2n = 48, ♂) as a result of chromosome doubling of diploid hybrid embryos (AB, 2n = 74) by inhibition of the first cleavage^21^. Unexpectedly, abnormal chromosome behaviour during meiosis, but not bivalent pairing, occurred in 4 nF_1_ and resulted in a generation of gametes with different genetic compositions, including allotetraploid (AABB), autotriploid (AAA), and autodiploid (AA) gametes^22^,^23^. Thus, we obtained autotetraploids (AAAA, 4n = 200) in the self-cross progenies of 4 nF_1_, and allopentaploid hybrids (5 nH) (AABBB, 5n = 172) in backcross progenies of 4 nF_1_ (♀) × BSB (♂)^21^,^24^. In addition, gynogenetic allotetraploids (4 nG) (AABB, 4n = 148) were obtained by artificial gynogenesis from eggs of the 4 nF_1_ that were activated with UV-treated sperm of BSB, but not subject to treatment for doubling the chromosome number^22^. Because the progenitors for these allopolyploids are known, we can precisely determine the timing and processes of genomic changes after polyploidisation. Thus, these allopolyploids provide a model system to study early chromosomal evolution after polyploidisation.

Cytogenetics studies using fluorescence *in situ* hybridisation (FISH) have reported chromosomal changes in many polyploid species^25^,^26^,^27^,^28^,^29^. To further understand allopolyploid genome evolution in a broad context, we used the first generation 4 nF_1_ hybrids and their backcross progenies to explore potential genomic changes on polyploidisation. We determined the response of two dispersed chromosomal loci (5S rDNA and centromere) that may be particularly important for *de novo* allopolyploidy, because they are likely to be most vulnerable to genetic changes after polyploidisation and (or) hybridisation^29^,^30^,^31^. Our results revealed rapid genomic changes occurred in the first generations after polyploidisation, and indicated instability of the newly established allotetraploid genome. The findings of this study provide new insights into chromosomal evolution in vertebrates.

## Results

### Organisation of 5S rDNA unit

**Figure 1:**
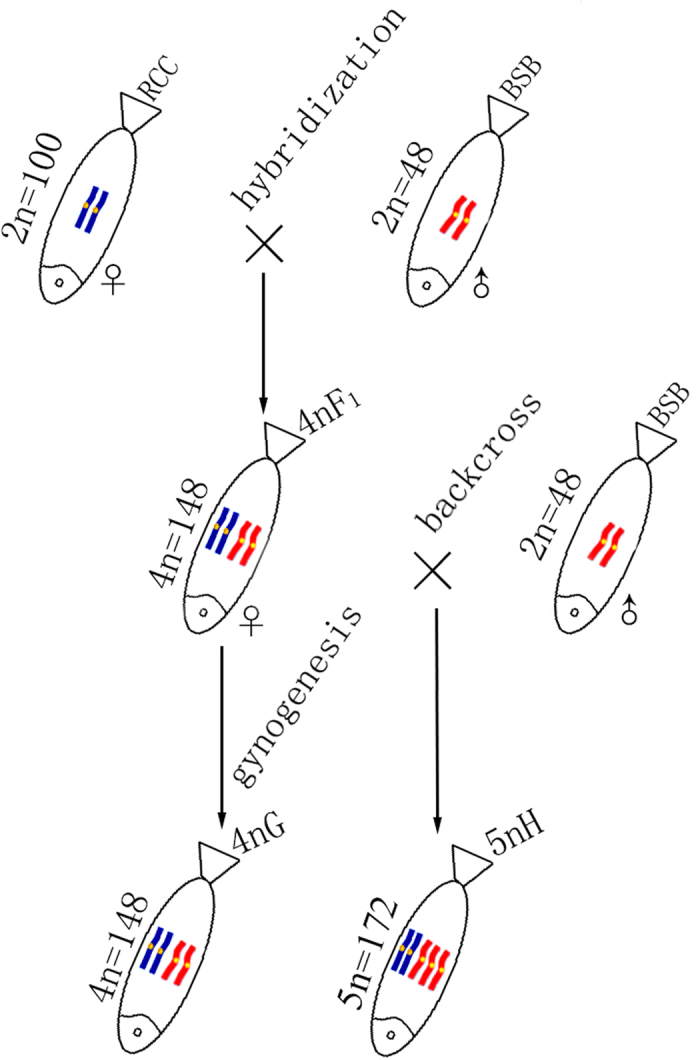
Formation of experimental fish hybrids. The parental origin of chromosomes is marked by blue and red. RCC, *Carassius auratus red var.*; BSB, *Megalobrama amblycephala*; 4 nF_1_, allotetraploid hybrid; 4 nG, allotetraploid gynogenetic hybrid; 5 nH, allopentaploid hybrid.

**Figure 2:**
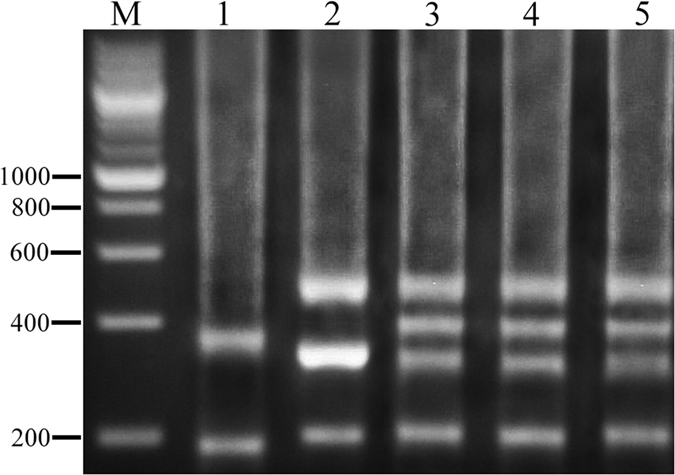
DNA fragments amplified from RCC, BSB, 4 nF_1_, 4 nG, and 5 nH. M, DNA ladder markers (200-bp increments); lane 1, DNA from BSB; lane 2, DNA from RCC; lane 3, DNA from 4 nF_1_; lane 4, DNA from 4 nG; lane 5, DNA from 5 nH. RCC, *Carassius auratus red var.*; BSB, *Megalobrama amblycephala*; 4 nF_1_, allotetraploid hybrid; 4 nG, allotetraploid gynogenetic hybrid; 5 nH, allopentaploid hybrid.

Formation and genetic compositionof experimental fish is showed in Fig. 1. DNA fragments were amplified from BSB, RCC, 4 nF_1_, 4 nG, and 5 nH using the 5SP1 and 5SP2R primers. Agarose gel electrophoresis identified three PCR fragments (approximately 200, 340, and 500 bp) from RCC, two PCR fragments (approximately 200 and 370 bp) from BSB, and four PCR fragments (approximately 200, 340, 400, and 500 bp) from each of 4 nF_1_, 4 nG, and 5 nH (Fig. 2).

A total of 340 clones were sequenced to examine the different patterns of the 5S rDNA sequences; 60 clones were from RCC, 40 clones were from BSB, and 80 clones were from each of the 4 nF_1_, 4 nG, and 5 nH hybrids (Table 1). Based on the BLASTn analysis, all the sequences from all five hybrids were confirmed as 5S rDNA repeat units. The 5S rDNA sequences from RCC fell into three distinct families (designated class I: 203 bp; class II: 340 bp; and class III: 477 bp), while the 5S rDNA sequences from BSB formed one family (designated class IV: 188 bp). All three RCC-derived families (class I, class II and class III) were detected in 4 nF_1_ and 4 nG, while the BSB-derived family (class IV) was not found in 4 nF_1_ and 4 nG. All four classes were detected in 5 nH (Table 1).

### Southern blot hybridisation

**Figure 3:**
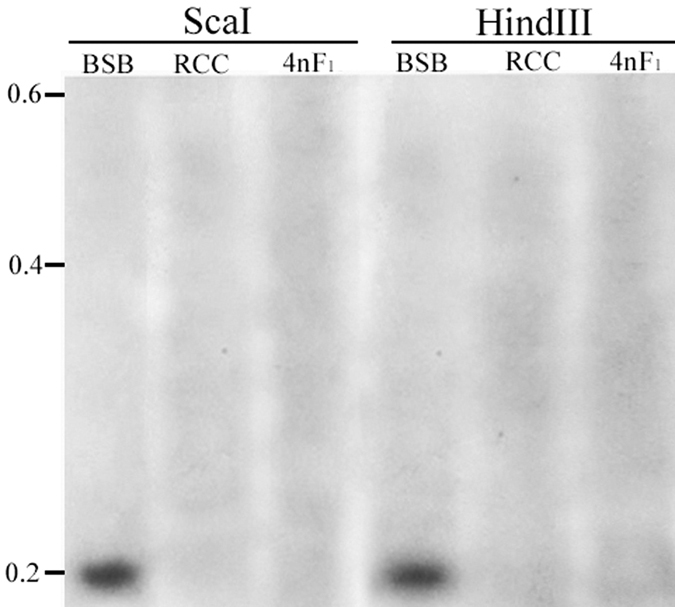
Southern blot hybridisation with the 5S rDNA sequence from BSB as a probe. *Hind*III and *Sca*I were used to digest the genomic DNA of BSB, RCC, and 4 nF_1_. Positive hybridisation was detected in the genomic DNA of BSB, but not in the genomic DNA of RCC and the 4 nF_1_ hybrid. Molecular weight makers (kb) are shown on the left. BSB, *Megalobrama amblycephala*; RCC, *Carassius auratus red var.*; 4 nF_1_, allotetraploid hybrid.

Genomic DNA from RCC, BSB and 4 nF_1_ was digested with *Hind*III and *Sca*I. Southern hybridisation was performed using the 5S rDNA sequence from BSB as a probe. This probe hybridised with the genomic DNA from BSB, but not with the genomic DNA from RCC and 4 nF_1_ (Fig. 3). This result implies that the paternal 5S rDNA cluster is completely deleted in 4 nF_1_.

### Fluorescence *in situ* hybridisation

**Figure 4:**
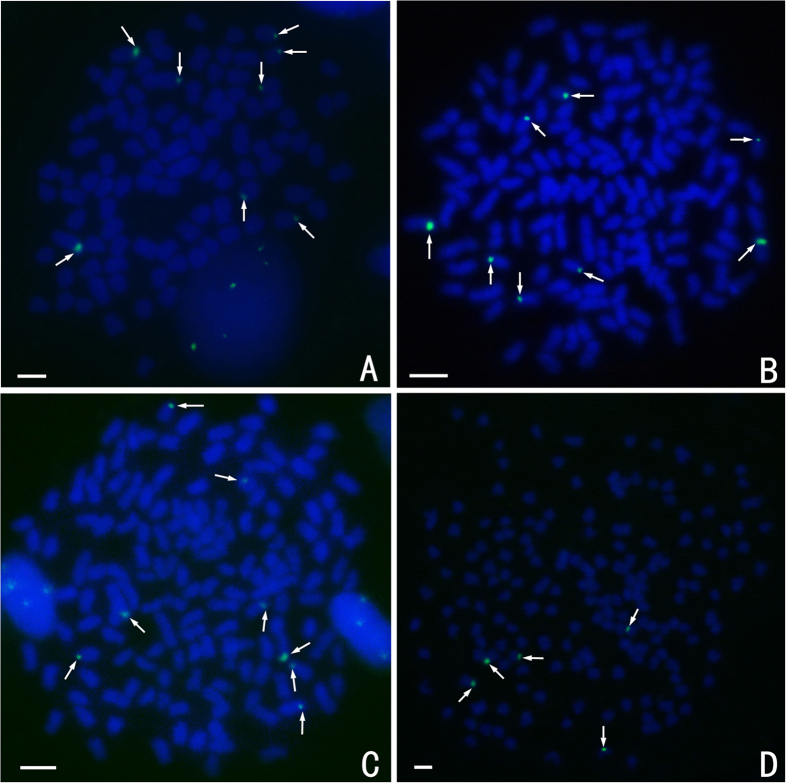
FISH hybridisation signals in the metaphase chromosomes of RCC, 4 nF_1_, 4 nG, and 5 nH with class I (203 bp) 5S rDNA as a probe. The white arrows indicate the 5S rDNA gene loci. The eight 5S rDNA gene loci in RCC (**A**), 4 nF_1_ (**B**), and 4 nG (**C**), and the five 5S rDNA gene loci in 5 nH (**D**) are shown. Bars in (**A**–**D**): 3 μm. RCC, *Carassius auratus red var.*; 4 nF_1_, allotetraploid hybrid; 4 nG, allotetraploid gynogenetic hybrid; 5 nH, allopentaploid hybrid.

FISH hybridisation of the RCC-derived class I (203 bp, GenBank: GQ485555) 5S rDNA gene probe to the RCC and BSB metaphase chromosomes yielded eight 5S rDNA gene loci in RCC (Fig. 4A; Table 2), but none in BSB (Table 2). It was expected that the eight 5S rDNA loci will also be present in the 4 nF_1_, 4 nG, and 5 nH hybrids because they were derived from RCC. However, we found that, while all eight 5S rDNA gene loci detected in the metaphase chromosomes of 4 nF_1_ and 4 nG and were similar to the eight RCC loci (Fig. 4B,C; Table 2), only five of the 5S rDNA gene loci were detected in the 5 nH metaphase chromosomes (Fig. 4D; Table 2).

**Figure 5:**
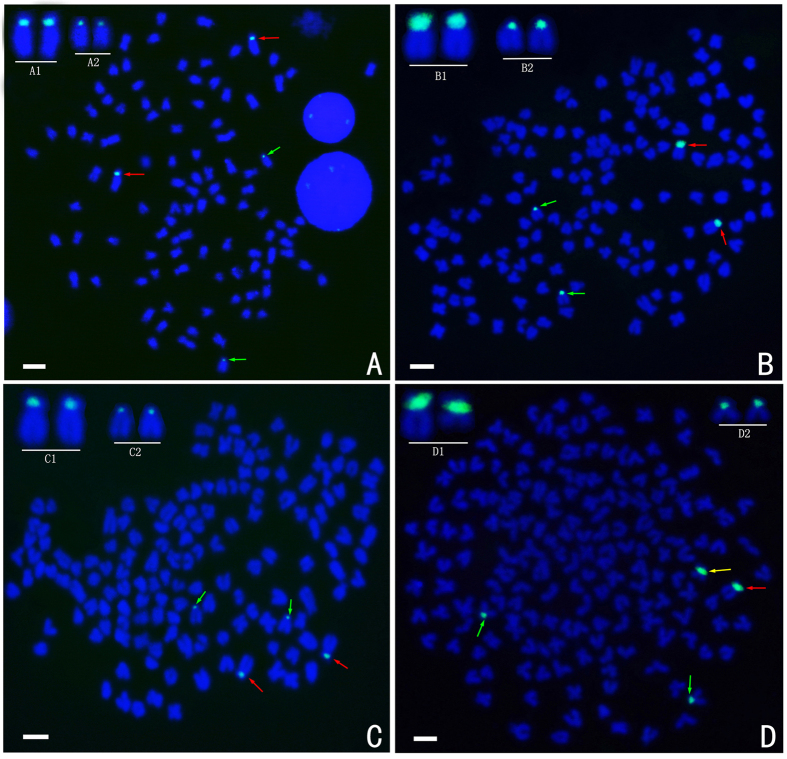
FISH hybridisation signals in the metaphase chromosomes of RCC, 4 nF_1_, 4 nG, and 5 nH with class II (340 bp) 5S rDNA as a probe. The red arrows indicate the large 5S rDNA gene loci and the green arrows indicate the small 5S rDNA gene loci. The two big and two small 5S rDNA gene loci in RCC (**A**), 4nF_1_ (**B**), and 4 nG (**C**) are shown. A1 in (**A**) B1 in (**B**) and C1 in (**C**) indicate that the large 5S rDNA gene loci were located on homologous submetacentric chromosomes. A2 in (**A**) B2 in (**B**) and C2 in (**C**) indicate that the small 5S rDNA gene loci were located on homologous subtelocentric chromosomes. (**D**) The two large 5S rDNA gene loci (red and yellow arrows) and two small 5S rDNA gene loci (green arrows) in 5 nH are shown. D1 indicates that the large 5S rDNA gene loci were located on a submetacentric chromosome (red) and a metacentric chromosome (yellow). D2 indicates that the small 5S gene loci were located on homologous subtelocentric chromosomes; Bars in (**A**–**D**) 3 μm. RCC, *Carassius auratus red var.*; 4 nF_1_, allotetraploid hybrid; 4 nG, allotetraploid gynogenetic hybrid; 5 nH, allopentaploid hybrid.

FISH hybridisation of the class II (340 bp, GenBank: GQ485556) 5S rDNA gene probe to the RCC and BSB metaphase chromosomes yield four 5S rDNA gene loci in RCC (Fig. 5A; Table 2), but none in BSB (Table 2). The chromosomal locus map for RCC revealed two large 5S rDNA gene loci on homologous submetacentric chromosomes, and two small 5S rDNA gene loci on homologous subtelocentric chromosomes (Fig. 5A). Similarly, as expected, two large and two small 5S rDNA gene loci were found on homologous submetacentric chromosomes and homologous subtelocentric chromosomes, respectively, in 4 nF_1_ (Fig. 5B) and 4 nG (Fig. 5C). Unexpectedly, in 5 nH, one large 5S rDNA gene locus was located on a submetacentric chromosome and another was located on a metacentric chromosome (Fig. 5D), suggesting that these two large 5S rDNA gene loci were not located on homologous chromosomes.

**Figure 6:**
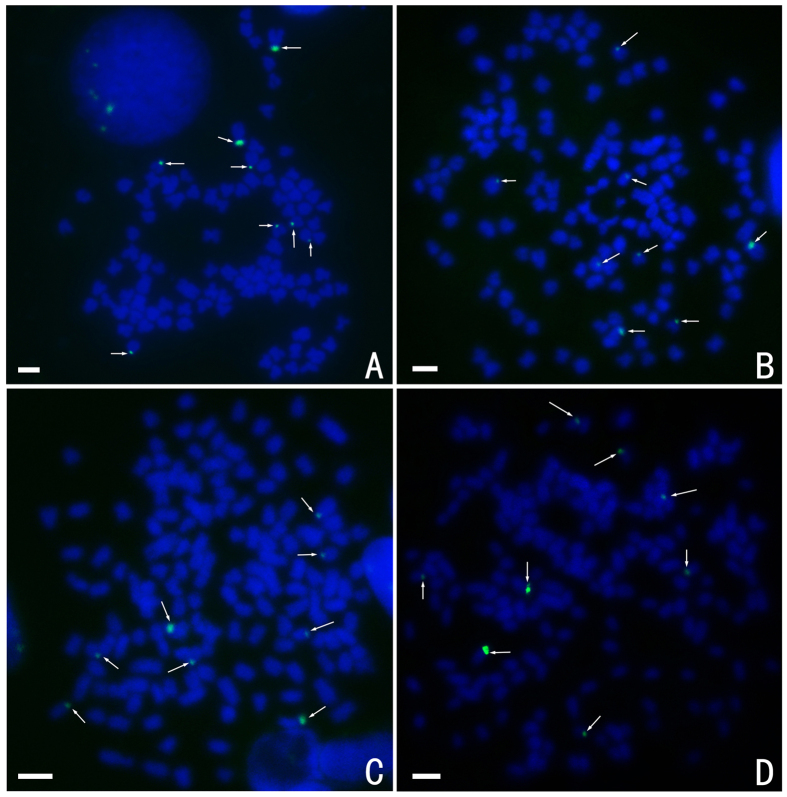
FISH hybridisation signals in the metaphase chromosomes of RCC, 4 nF_1_, 4 nG, and 5 nH with class III (477 bp) 5S rDNA as a probe. The white arrows indicate the 5S rDNA gene loci. The eight 5S gene loci in RCC (**A**), 4 nF_1_ (**B**), 4 nG (**C**), and 5 nH (**D**) are shown. Bars in (**A**–**D**): 3 μm. RCC, *Carassius auratus red var.*; 4 nF_1_, allotetraploid hybrid; 4 nG, allotetraploid gynogenetic hybrid; 5 nH, allopentaploid hybrid.

FISH hybridisation of the class III (477 bp, GenBank: GQ485557) 5S rDNA gene probe to the RCC and BSB metaphase chromosomes yield eight 5S gene loci in RCC (Fig. 6A; Table 2), but none in BSB (Table 2). As expected, the eight RCC-derived 5S rDNA gene loci were detected in the metaphase chromosomes of 4 nF_1_, 4 nG and 5 nH and were similar to the loci in RCC (Fig. 6B–D; Table 2).

**Figure 7:**
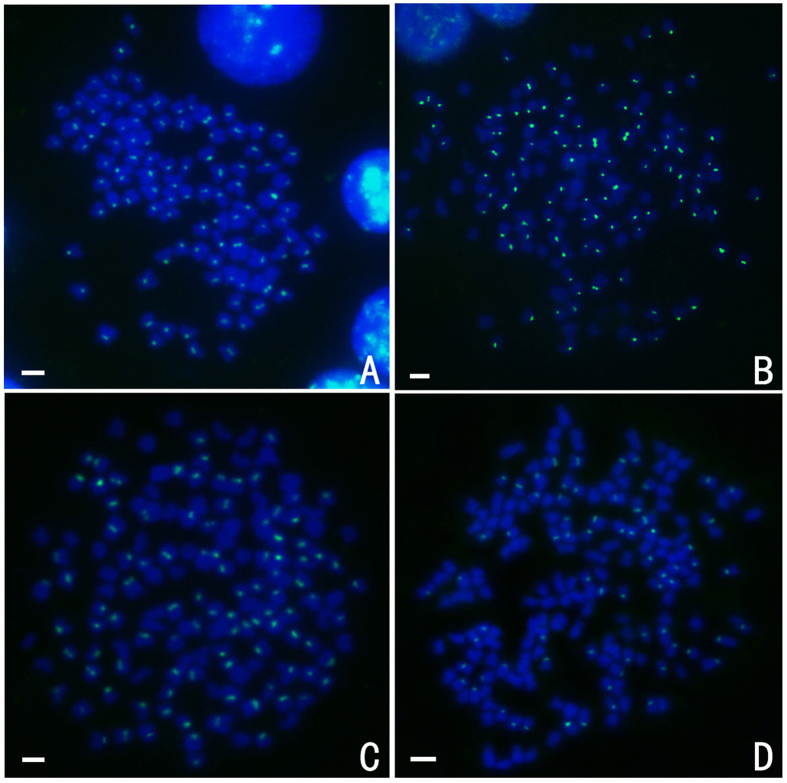
FISH hybridisation signals in the metaphase chromosomes of RCC, 4 nF_1_, 4 nG, and 5 nH with the centromere probe. The centromere probe hybridised to 100 chromosomes in RCC (**A**), 4 nF_1_ (**B**), and 4 nG (**C**), and to 69 chromosomes in 5 nH (**D**). Bars in (**A**–**D**): 3 μm. RCC, *Carassius auratus red var.*; 4 nF_1_, allotetraploid hybrid; 4 nG, allotetraploid gynogenetic hybrid; 5 nH, allopentaploid hybrid.

The RCC-derived centromere probe (GenBank: JQ086761) hybridised to 100 chromosomes in RCC (Fig. 7D), but none in BSB (Table 2). This species-specific centromere probe also hybridised to 100 metaphase chromosomes in 4 nF_1_ and 4 nG, as expected, but only about 65 to 70 RCC-derived centromere loci were detected in 5 nH metaphase chromosomes, rather than the expected 100 loci.

## Discussion

Polyploidisation may increase genomic variation rates and is important for the formation in new polyploid species^4^. Evidence for genomic variations, including fragment loss, chromosomal rearrangement, and rDNA loci changes have been reported in both synthesised polyploid^7^,^27^,^32^ and natural polyploid species^25^,^26^. Genomic variations usually occur in the early generations after polyploidisation, possibly reflecting instability in newly established polyploid genomes^26^,^27^. The results of the present study support previous observations that genomic changes occur in newly established polyploid genomes, and reveal that these changes can begin as early as the first generation after polyploidisation.

Because of incompatibility between homeologous chromosomes, hybridization can boost genomic change^33^,^34^. The frequency of genomic change has been associated with divergence of the diploid parental genomes^7^. The 4 nF_1_ hybrid was formed by combining the two diploid genomes from RCC and BSB, two fish species in the family *Cyprinidae*, that belonged to different subfamilies (*Cyprininae* and *Cultrinae*)^21^, implying RCC and BSB are genetically distinct. In 4 nF_1_, not only was the paternal 5S rDNA unit deleted entirely, but so were the paternal *sox*^21^,^35^ and *hox* (unpublished data) gene families, suggesting that a large number of genomic changes had occurred in the newly established allotetraploid genome. A variety of genomic changes can result in diploid-like chromosome pairing, which has been reported to prevent meiotic irregularities and improve the efficiency of gamete production in polyploid species^16^,^36^. However, there is still no direct evidence that large numbers of genomic variations or unstable individuals are selected for during the establishment of polyploid species^27^,^37^,^38^,^39^. In previous study, we found that diploid-like chromosome pairing was not restored in 4 nF_1_^22^,^23^. We speculated that mass deletion of paternal genetic material gave rise to excessive genomic modification in 4 nF_1_, which prevented diploid-like chromosome pairing, and resulted in weak fertility and the generation of gametes with a different genetic composition. To avoid extinction, the unstable 4 nF_1_ individuals may have entered a novel evolutionary trajectory by abnormal meiosis, and produced diploid gamete with two sets of RCC-derived chromosomes. Thus, unexpectedly, we obtained better fertile autotetraploids among the progenies of 4 nF_1_, and successfully established an autotetraploid fish line (F_2_–F_9_)^24^.

In some cases, it has been shown that hybridization had more effect on the change in genomic and gene expression than polyploidization^40^,^41^. In our study, 4nG result from genome doubling of germ cell, 5 nH was obtained by hybridization of 4nF_1_ (♀) × BSB. Thus, the 5S rDNA units and chromosomal loci (5S rDNA and centromere) remained intact in the 4nG genome, but obvious variations were found in 5 nH. In addition, our data also revealed the elimination of the entire paternal 5S rDNA unit, and stabilisation of the maternal 5S rDNA units and chromosomal loci in the allotetraploid hybrids, implying that the paternal genome underwent greater polyploidisation-associated modifications than the maternal genome. Similar findings have been reported in polyploid plants^30^,^42^,^43^. The nucleo-cytoplasmic hypothesis might be an explanation for the apparent paternal genome lability. This hypothesis predicts that the paternal genome of a newly formed allopolyploid evolves most rapidly because the maternal cytoplasmic background leads to paternal genome instabilities^7^. However in 5 nH, the newly established maternal allotetraploid genome showed obvious variations in chromosomal loci, while the parental 5S rDNA units remained intact, suggesting that the maternal genome was more unstable than the parental genome. These results are opposite to those predicted by the nucleo-cytoplasmic hypothesis. We speculate that the genetic variations in the maternal chromosomal loci may be attributed to instability of the newly established allotetraploid genome. Further, the newly established allotetraploid genome consists of the BSB-derived genome, which may hinder or reduce the influence of the cytoplasmic background on the instability of the BSB-derived paternal genome.

## Methods

### Animals and crosses

All experiments, performed from 2012–2015, were approved by the Animal Care Committee of Hunan Normal University. The Administration of Affairs Concerning Animal Experimentation guidelines stated approval from the Science and Technology Bureau of China. The methods were carried out in accordance with the approved guidelines. Experimental individuals were fed in a pool with suitable illumination, water temperature, dissolved oxygen content, and adequate forage in the Engineering Center of Polyploidy Fish Breeding of the National Education Ministry located at Hunan Normal University, China. Approval from the Department of Wildlife Administration is not required for the experiments conducted in this paper. Fish were deeply anesthetized with 100 mg/L MS-222 (Sigma-Aldrich) before dissection.

The 4nF_1_ hybrids (AABB, 4n = 148) of RCC (AA, 2n = 100, ♀) × BSB (BB, 2n = 48, ♂) were produced during the reproductive seasons (April to June) in 2012, 2013, and 2014. During the reproductive season of 2014, the gynogenetic allotetraploid hybrids (4 nG) (AABB, 4n = 148) were obtained by artificial gynogenesis from the eggs of the 4 nF_1_ that were activated with UV-treated sperm of BSB without treatment for doubling the chromosomes. During the reproductive season of 2015, 5 nH (AABBB, 5n = 172) was obtained in backcross progenies of 4nF_1_ (♀) × BSB (♂).

### PCR amplification and sequencing

One pair of primers (5SP1: 5′-GCTATGCCCGATCTCGT CTGA-3′ and 5SP2R: 5′-CAGGTTGGTATGGCCGTAAGC-3′) was designed and synthesised to amplify the 5S rDNA repeats directly from genomic DNA by PCR. The PCR reactions and sequencing were performed as described by Qin *et al*.^44^. Sequences were analysed using ClustalW software (http://www.ebi.ac.uk/Tools/msa/clustalw2/).

### Southern blot hybridisation

Genomic DNA (10 mg) from all the samples from RCC, BSB and 4nF_1_ was completely digested with the restriction endonucleases *Hind*III and *Sca*I, submitted to 0.8% agarose gel electrophoresis, and transferred onto Hybond-N1 membrane^45^. The 5S rDNA sequences were labelled with Dig-11-dUTP (Roche), which was used as a probe, and hybridised with the filter-immobilised DNA. Hybrid signal detection was performed with a DIG detection kit II (Innogent, China).

### Fluorescence *in situ* hybridisation

Chromosome preparation was carried out on the kidney tissues of all samples, according to the procedures reported by Liu *et al*.^21^ The FISH probes for the 5S gene and species-specific centromere were amplified by PCR using 5SP1 and 5SP2R primer, and the primer 5′-TTCGAAAAGAGAGAATAATCTA-3′ and 5′-AACTCGTCTAAACCCGAACTA-3′, respectively. The FISH probes were produced by Dig-11-dUTP labelling (using a nick translation kit; Roche, Germany) of the purified PCR products. FISH was performed according to the method described by He *et al*.^46^ For each type of fish hybrid, 200 metaphase spreads (20 metaphase spreads in each sample) of the chromosomes were analysed.

## Additional Information

**How to cite this article**: Qin, Q. *et al*. Rapid genomic changes in allopolyploids of *Carassius auratus red var*. (♀) × *Megalobrama amblycephala* (♂). *Sci. Rep.* **6**, 34417; doi: 10.1038/srep34417 (2016).

## Figures and Tables

**Table 1:** Organisation of 5S rDNA units in five fish types.

Fish type^a^	No. of sequenced clones	Type of 5S rDNA units			
		class I (203 bp)	class II (340 bp)	class III (477 bp)	class IV (188 bp)
RCC	60	+	+	+	−
BSB	40	−	−	−	+
4 nF_1_	80	+	+	+	−
4 nG	80	+	+	+	−
5 nH	80	+	+	+	+

^a^RCC, *Carassius auratus red var.*; BSB, *Megalobrama amblycephala*; 4 nF_1_, allotetraploid hybrid; 4 nG, allotetraploid gynogenetic hybrid; 5 nH, allopentaploid hybrid.

**Table 2:** Examination of hybridising signals by FISH in five fish types.

Fish type^a^	No. of Fish	No. of metaphase	203 bp	263 bp	477 bp	304 bp	
			No. of loci	No. of loci	No. of loci	No. of big loci	No. of small loci
RCC	10	200	8	100	8	2	2
BSB	10	200	0	100	0	0	0
4 nF_1_	10	200	8	100	8	2	2
4 nG	10	200	8	100	8	2	2
5 nH	10	200	4–5	65–70	8	2	2

^a^RCC, *Carassius auratus red var.*; BSB, *Megalobrama amblycephala*; 4 nF_1_, allotetraploid hybrid; 4 nG, allotetraploid gynogenetic hybrid; 5 nH, allopentaploid hybrid.
